# Potential Beneficial Effects of Cytomegalovirus Infection after Transplantation

**DOI:** 10.3389/fimmu.2018.00389

**Published:** 2018-03-01

**Authors:** Nicolle H. R. Litjens, Lotte van der Wagen, Jurgen Kuball, Jaap Kwekkeboom

**Affiliations:** ^1^Department of Internal Medicine, Nephrology and Transplantation, Erasmus MC, University Medical Center, Erasmus University Rotterdam, Rotterdam, Netherlands; ^2^Laboratory of Translational Immunology, Department of Hematology, University Medical Center Utrecht, Utrecht, Netherlands; ^3^Department of Gastroenterology and Hepatology, Erasmus MC, University Medical Center, Erasmus University Rotterdam, Rotterdam, Netherlands

**Keywords:** cytomegalovirus infections, hematopoietic stem cell transplantation, solid organ transplantation, leukemia, tolerance

## Abstract

Cytomegalovirus (CMV) infection can cause significant complications after transplantation, but recent emerging data suggest that CMV may paradoxically also exert beneficial effects in two specific allogeneic transplant settings. These potential benefits have been underappreciated and are therefore highlighted in this review. First, after allogeneic hematopoietic stem cell transplantation (HSCT) for acute myeloid leukemia (AML) using T-cell and natural killer (NK) cell-replete grafts, CMV reactivation is associated with protection from leukemic relapse. This association was not observed for other hematologic malignancies. This anti-leukemic effect might be mediated by CMV-driven expansion of donor-derived memory-like NKG2C^+^ NK and Vδ2^negγδ^ T-cells. Donor-derived NK cells probably recognize recipient leukemic blasts by engagement of NKG2C with HLA-E and/or by the lack of donor (self) HLA molecules. Vδ2^negγδ^ T cells probably recognize as yet unidentified antigens on leukemic blasts via their TCR. Second, immunological imprints of CMV infection, such as expanded numbers of Vδ2^negγδ^ T cells and terminally differentiated TCRαβ^+^ T cells, as well as enhanced NKG2C gene expression in peripheral blood of operationally tolerant liver transplant patients, suggest that CMV infection or reactivation may be associated with liver graft acceptance. Mechanistically, poor alloreactivity of CMV-induced terminally differentiated TCRαβ^+^ T cells and CMV-induced IFN-driven adaptive immune resistance mechanisms in liver grafts may be involved. In conclusion, direct associations indicate that CMV reactivation may protect against AML relapse after allogeneic HSCT, and indirect associations suggest that CMV infection may promote allograft acceptance after liver transplantation. The causative mechanisms need further investigations, but are probably related to the profound and sustained imprint of CMV infection on the immune system.

## Introduction

While the positive impact of host–microbiota interaction on human health is being extensively studied in recent years, possible beneficial effects of life-long persistent viruses on human health remain a whole new world to explore. One of the most prevalent viruses among humans is cytomegalovirus (CMV). The sero-prevalence of CMV ranges from 30 to 100% depending on socioeconomic and ethnic background. CMV generally remains quiescent in healthy individuals, but can cause severe disorders in immunocompromised individuals, such as patients after hematopoietic stem cell transplantation (HSCT) or solid organ transplantation (SOT). Paradoxically, accumulating recent evidence suggests that CMV infection after transplantation may also have beneficial effects, particularly in protection against leukemic relapse following HSCT for acute myeloid leukemia (AML) and in promoting graft acceptance after liver transplantation (LTx). Recent research has shed first light on potential immunological mechanisms behind these surprising beneficial effects. Here, we discuss recent evidence for these two potential benefits of CMV infection after transplantation and emerging insights into the immunological mechanisms that may be involved.

## Anti-Leukemic Effect of CMV Reactivation after Allogeneic HSTC for AML

Cytomegalovirus reactivation is a frequent and major complication after HSCT, causing a variety of organ-specific diseases, including pneumonia, encephalitis, and gastrointestinal disease. Prior to the age of prophylactic and pre-emptive treatment of CMV reactivation, CMV pneumonia was the most common infectious cause of death after HSCT. Despite, advances in diagnostic techniques and treatment strategies, CMV seropositivity remains to be associated with inferior outcome, especially after myeloablative HSCT (1–3). However, paradoxical observations show that CMV reactivation may protect against leukemic relapse after allogeneic HSCT for AML go back to the mid-1980s (4), and have been confirmed in a series of recent studies which we summarize in Table 1.

**Table 1:** Summary of recent studies on the association of post-transplant Cytomegalovirus (CMV) replication and relapse of hematological malignancies after HSCT.

Study (Ref.)	Effect	Patients	Adults/pediatric patients	Myeloablative pre-conditioning	T/NK-depleted graft	Antibody-based in vivo T-cell depletion	Donors	Stem cell source	CMV dete-ction	Endpoint	Relapse rate			Effect of CMV reactivation on AML relapse	Comments
											With CMV reactivation	Without CMV reactivation	*P*-value		
Elmaagacli et al. (5)	Positive in AML	AML *n* = 266	Adults	All	No	No	Sibs 118 (44%); MUD 148 (56%)	BM 45 (17%); PBSC 221 (83%)	pp65 anti-genemia	Cumulative incidence of AML relapse at 10 years after HSCT = 33% (95% CI, 27–40%)	10-year CIR AML 9%	10-year CIR AML 42%	<0.0001	^a^CMV infection: HR = 0.2, 95% CI = 0.1–0.4, *P* < 0.0001	

Manjappa et al. (7)	Positive in AML	AML *n* = 264	Adults	206 (78%)	no	46 (17%) ATG	MRD 108 (41%); MUD 156 (59%)	BM 23 (9%); PBSC 240 (91%)	PCR	Cumulative incidence of AML relapse at 6 years after HSCT = 43%	6-year CIR AML 38.9%	6-year CIR AML 59%	0.03	^a^CMV infection: HR = 0.53,95% CI = 0.33–0.83, *P* = 0.015	Effect restricted to patients receiving myelo-ablative conditioning

Jang et al. (8)	Positive in AML	AML *n* = 74	Median age 35; range 15–59 years	68 (92%)	Not men-tioned	11 (15%) A TG or alemtu-zumab	MRD 31 (42%); MUD 43 (58%)	BM 5 (7%); PBSC 69 (93%)	PCR	Cumulative incidence of AML relapse at 5 years after HSCT = 31%	Patient numbers not mentioned			^a^CMV infection: HR = 0.21,95% CI = 0.08–0.54, *P* = 0.001	

Green et al. (6)	Positive in AML	AML *n* = 761ALL *n* = 322CML *n* = 646Lymphoma *n* = 254MDS n = 371	2,306 adults/260 children	659 (87%) of AML patients	39 (5%) of AML patients	Not mentioned	Sibs 397 (52%); MUD 351 (46%); haplo 12 (2%)	BM 301 (40%); PBSC 460 (60%)	pp65 anti-genemia	Cumulative incidence of AML relapse at 1 year after HSCT = 25.2%	1-year CIR AML 26.5%	1 year CIR AML 32.7%	0.19	^a^CMV infection: HR = 0.56, 95% CI = 0.3–0.9, *P* = 0.02^c^	Effect restricted to AML patients and no effect on overall mortality

Takenaka et al. (9)	Positive in AML	AML *n* = 1836ALL *n* = 911CML *n* = 223MDS *n* = 569	Median age 46; range: 16–74 years	1381 (75%) of AML patients	No	No	MRD 989 (54%); MUD 847 (46%)	BM 1267 (69%); PBSC 569 (31%)	pp65 anti-genemia	Cumulative incidence of AML relapse at 5 years after HSCT = 26.5%	5-year CIR AML 22.4%	5-year CIR AML 29.6%	<0.01	^a^CMV infection: HR = 0.77, 95%C = 0.59–0.99, *P* = 0.04	Effect restricted to AML patients

Bao et al. (10)	Positive in AML sub-group	AML*n* = 227	Adults + children, median age 35; range 2–63 years	All	No	117 (52%) ATG	Sibs 110 (49%), MUD 57 (25%), haplo 60 (26%)	BM 50 (22%), PBSC 125 (55%), BM + PBSC 52 (23%)	PCR	Cumulative incidence of AML relapse at 3 years after HSCT = 26%	3-year CIR AML 22%Subgroup: non ATGpatients 3-year CIRAML 8.9%	3-year CIR AML 29.7%Subgroup: non ATGpatients 3-year CIR AML26.7%	0.237 0.016	^a^CMV infection: H R = 0, 28, 95% CI = 0.1–0.79, *P* = 0.016	Effect restricted to subgroup of patients NOT receiving A TG in conditioning (*n* = 110)

Ramanathan et al. (14)	Positive trend in AML	AML *n* = 925ALL *n* = 759	Adults + children, median age 28; range 1–79 years	680 (74%) of AML patients	Not men-tioned	Part of patients, number not mentioned	CB	CB	Unknown	Cumulative incidence of AML relapse at 3 years after HSCT = 35%	Patient numbers not mentioned			^a^CMV infection: HR = 0.895% CI = 0.62–1.04, *P* = 0.097	Trend restricted to AML patients

Teira et al. (12)	Negative in AML	AML *n* = 5310ALL *n* = 1883CML *n* = 1079MDS *n* = 1197	Median age 48; range 1–83 years	3,809/5310 (72%)	149/5,310 (3%)	1,439/5,310 (27%)	Sibs 4071 (43%); MUD 3481 (37%)	BM 2475 (26%); PBSC 6994 (74%)	Unknown	Cumulative incidence of AML relapse at 3 years after HSCT = 38%	Patient numbers not mentioned			^a^CMV infection: HR = 0.97 95%CI = 0.86–1.1, *P* = 0.6 (all AML pts)	Also no effect in ALL, CML, MDS

Ito et al. (15)	Positive in CML	CML *n* = 110	Adults + children, median age 36; range 13–69 years	97 (88%)	97 (88%)	No	Sibs 110 (100%)	BM 27 (25%), PBSC 83 (75%)	pp65 anti-genemia and PCR	Cumulative incidence of CML relapse = 49% relapse after median follow-up of 6–2 years	Patient numbers not mentioned			^a^CMV infection: HR = 0.533,95% CI = 0.29–0.99, *P* = 0.045	

Koldehoff et al. (17)	Positive in NHL	B-cell lymphoma *n* = 94T- and NK cell neoplasms *n* = 42	Adults	107 (79%)	Not men-tioned	57 (42%) ATG	Sibs 36 (26%), MUD 80 (59%), MUD-MM 20 (15%)	BM 11 (8%); PBSC 125 (92%)	pp65 antigenemia and PCR (after June 2011)	Cumulative incidence of NHL relapse at 5 years after HSCT = 31%	5-year CIR NHL 22%	5-year CIR NHL 38%	< 0.013	^a^CMV infection: HR = 0.29, 95%CI = 0.11–0.76, *P* < 0.014	

Mariotti et al. (16)	Negative in NHL	B-cell lymphoma *n* = 265	Adults	75 (26%)	No	124 (45%) ATG or alemtu-zumab	Sibs 147 (55%), MUD 50 (19%), MUD-MM 68 (26%)	BM 26 (10%), PBSC 239 (90%)	pp65 antigene-mia	Cumulative incidence of B-cell lymphoma relapse at 5 years after HSCT = 42%	5-year CIR NHL 34%	5-year CIR NHL 50%	0.42	^a^CMV infection: HR = 1.0, 95% CI = 0.6–1.6; *P* = 0.9	

*ALL, acute lymphoblastic leukemia; AML, acute myeloid leukemia; BM, bone marrow; CB, cord blood; CIR, cumulative incidence of relapse; CML, chronic myeloid leukemia; D/R, donor/recipient; haplo, haploidentical donor; MDS, myelo-dysplastic syndrome; MM, mismatched; MRD, matched related donor allogeneic HSTC; MUD, matched unrelated donor allogeneic HSCT; NHL, non Hodgkin lymphoma; PBSC, peripheral blood stem cells; sib, sibling donor allogeneic HSCT*.

*^a^Multivariate analysis*.

*^b^Center for International Blood and Marrow Transplant (CIBMTR) database*.

*^c^Effect calculated on cumulative*.

In a homogeneous cohort of adult AML patients monitored by the pp65 antigenemia assay and treated with preemptive anti-CMV therapy, patients with early CMV replication after allo-HSCT had a significantly reduced risk to develop relapse within 10 years after transplantation (5). In a large cohort of allo-HSCT patients treated for different hematologic malignancies, Green et al. confirmed the anti-leukemia effect of early CMV replication detected by pp65 in AML patients, but did not observe such effect in acute lymphoblastic leukemia (ALL), chronic myeloid leukemia (CML), lymphoma, and myelodysplastic syndrome (MDS) patients (6). Interestingly, pre-transplant CMV seropositivity was associated with an increased risk of relapse, which was confirmed in another study (1). Therefore, not pre-transplant CMV serostatus, but actual CMV reactivation seems to contribute to the observed beneficial effect.

The association between early CMV replication after allo-HSCT and reduction of AML relapse risk was further confirmed by four independent recent studies from different countries (7–10). However, this association was not observed in patients who did not receive a myeloablative conditioning regimen (7) or were treated with an *in vivo* T-cell depleting therapy, such as ATG or alemtuzumab (10, 11). In three of these studies, CMV reactivation was determined by PCR. In contrast, one recent registry study, included 5,310 AML patients, showed no benefit of CMV reactivation for AML relapse risk after allo-HSCT (12). However, in this study 28% of AML patients did not receive myeloablative therapy and 27% of AML patients were treated by *in vivo* T/NK-cell depleting therapy. In addition, the methods for evaluation of CMV reactivation were unknown, which may have resulted in different definitions of CMV reactivation. A recent meta-analysis of 6 studies, including the recent registry study (12), with 8,511 AML patients who received mainly T-cell replete grafts and were not treated with T-cell depleting therapy, confirmed that CMV reactivation after allo-HSCT results in a substantial reduction of the risk of relapse (HR = 0.6, 95% CI = 0.43–0.84, *P* = 0.003) (13).

Thus, the evidence of a protective association between CMV replication and leukemic relapse in AML patients appears compelling, but only under specific transplantation conditions (7, 10, 11). However, it should be emphasized that only three studies reported an improved overall survival in AML patients with CMV replication after HSCT (5, 8, 10), while the majority of studies found that the anti-leukemic effect did not translate into improved survival. Indeed, other studies found either no difference in survival between patients with and without CMV replication (6, 7) or reported that CMV replication was associated with worse survival due to increased non-relapse mortality (9, 12).

The only evidence available in the cord blood transplantation setting is a recent registry study (14), which showed a trend to reduced AML relapse in patients with CMV reactivation. However, like in the registry study of Teira et al. (12), the methods used to detect CMV reactivation are unknown, and part of the patients received T-cell depleting therapy.

Whether CMV reactivation can protect against relapse after allo-HSCT for other hematological malignancies is controversial. While Ito et al. found a decreased risk of relapse in CML patients after CMV reactivation within 100 days after allo-HSCT (15), two other studies did not confirm this finding (6, 9). Most studies did not observe any beneficial effect of CMV reactivation after allo-HSCT for acute lymphoblastic leukemia (ALL), myelodysplastic syndrome (MDS), or lymphomas (6, 9, 14, 16), but a recent study by Koldehoff et al. reports a reduced relapse incidence in non-Hodgkin lymphoma (NHL) patients (17). Because of contradictory results reported for other hematological malignancies, results on associations between CMV reactivation and relapse after allo-HSCT derived from mixed populations of patients with different hematological malignancies are difficult to interpret (3, 18).

In addition, apart from one study where more severe (grade II–IV) acute GVHD was observed in patients with CMV reactivation (10), it was not associated with acute or chronic GVHD and remained an independent risk factor for AML relapse in multivariate analyses in which GVHD was included (Table S1 in Supplementary Material), however, this was not always reported. Available data, therefore, suggest that protection of AML relapse cannot be merely explained by an increased CMV-induced allogeneic immune response.

## Possible Mechanisms of Anti-Leukemic Effects of CMV after allo-HSCT for AML

**Figure 1:**
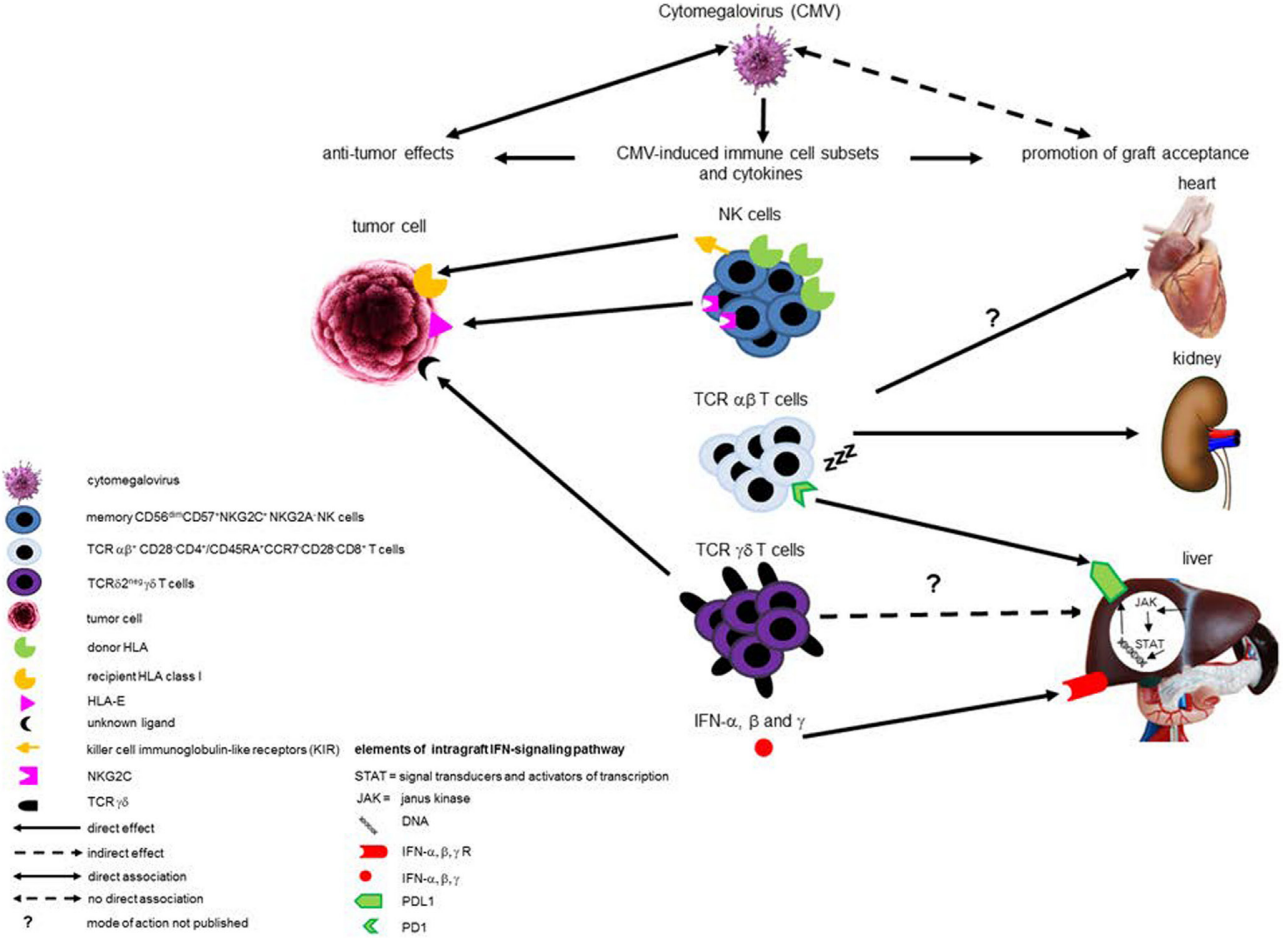
Schematic overview of (potential) beneficial effects of cytomegalovirus (CMV) infection after transplantation. This schematic overview illustrates the (potential) beneficial effects exhibited by different CMV-induced immune cell subsets and intra-graft IFN-signaling pathways after transplantation. Compelling evidence exists for anti-leukemic effects of CMV-induced donor-derived memory-like natural killer (NK) cells after hematopoietic stem cell transplantation (HSCT) in acute myeloid leukemia (AML) patients, and two mechanisms have been described. One involves enhanced expression of the activating NK-receptor NKG2C, at the expense of the inhibitory NKG2A, interacting with HLA-E expressed by AML blasts. The other mechanism involves the “missing self” principle, as recipient tumor cells do not express donor HLA class I and are, therefore, a target for killing by donor-derived NK cells as a result of lack of inhibition *via* donor HLA class I-recognizing inhibitory killer cell immunoglobulin-like receptors. CMV-induced TCRδ2^−^ γδ T cells have also been associated with anti-leukemic effects after HSCT, probably *via* recognition of an as yet unknown ligand by their TCR. Evidence implicating CMV-specific TCR αβ T cells in preventing AML relapse after HSCT is lacking. In addition, CMV-induced immune cell subsets have been associated with graft acceptance and liver-transplant tolerance. Evidence merely consists of associations and no detailed mechanistic insights are available yet. Induction of terminally differentiated TCRαβ T cells with low alloreactivity by CMV infection in various types of solid organ transplantations may be involved in development of graft acceptance. CMV-induced circulating TCRδ2^−^ γδ T cells are associated with liver transplant tolerance, but probably not functionally involved. Overexpression of NKG2C in peripheral blood is associated with both CMV infection and graft acceptance after liver transplantation, but whether a causal exists between NKG2C^+^ NK cells and graft acceptance is unknown. Apart from CMV-induced immune cell subsets, intra-graft IFN-α, β, and γ production, which can be induced by CMV, has been associated with liver transplant tolerance by induction of PD-L1 expression in the graft, thereby counteracting the host immune response.

Cytomegalovirus reactivation does not protect against AML relapse when T- and/or NK cells are depleted *in vivo* or *in vitro*, suggesting that CMV reactivation requires a reconstitution of donor-derived T cells and/or NK cells to reduce leukemic relapse (7, 10, 11). Although recently a direct pro-apoptotic effect of CMV on acute leukemia cell lines has been shown (19), CMV is generally thought to be non-cytolytic, but instead to protect infected cells from apoptosis in order to delay cell death and maintain viral replication. Therefore, the anti-leukemic effects of CMV infection after allo-HSCT for AML are probably mainly caused by cross-reactivity of CMV-stimulated innate and adaptive immune responses with cancer cells. CMV infection leaves a deep and life-long imprint on the human immune system. Two types of immune cells that are expanded during CMV infection have been postulated to be involved in CMV-induced protection against AML. These are natural killer (NK) cell and Vδ2^negγδ^ T cells (Figure 1).

A CMV reactivation after allogeneic HSCT induces a long-lasting expansion of, mainly donor-derived, memory-like NK cells, or CMV-adapted NK cells, with enhanced functional properties compared to conventional NK cells. This CMV-induced memory-like NK-cell population is characterized by low expressionof CD56, expression of CD57, lack of the inhibitory NKG2a receptor, and expression of the activating heterodimeric receptor CD94-NKG2C (20, 21). The memory-associated features of these CMV-induced NK include secondary expansion and enhanced capacity to produce IFN-γ upon CMV reactivation (20, 22, 23). Once induced, their expansion is not limited to CMV reactivation, as stimulation *via* the low affinity Fc receptor IIIa (CD16) by IgG, as well as pro-inflammatory cytokines, can contribute to the expansion, persistence, and functional properties of CMV-induced memory-like NK cells (21, 24, 25). The enhanced functional properties of CMV-induced donor-derived NKG2C^+^ memory NK cells compared to conventional NK cells are caused by epigenetic remodeling resulting in increased proliferative responses as well as cytokine production (21, 24, 26).Interestingly, expansion of these cells after HSCT is not only associated with protection from CMV reactivation (27), but also trended to be associated with a reduced rate of AML relapse (28).

The mechanism by which this NK-cell subset recognizes leukemic blasts may be related to the switch in expression from the inhibitory NKG2A to the activating NKG2C, both receptors for HLA-E, which is expressed on leukemic blasts. Alternatively, in partially HLA-mismatched HSCT, the anti-leukemic effect may be related to the general mechanism of NK-cell self-tolerance, which is mediated by inhibitory receptors, such as killer cell immunoglobulin-like receptors (KIR), that recognize self-MHC class I molecules. According to the “missing-self hypothesis,” recipient AML cells can be the target for cytotoxicity of donor-derived NKG2C^+^ NK cells, induced upon CMV infection, as they lack donor HLA molecules (23).

γδ T cells are involved in the first line of host immune defense to microbial pathogens and expanded in the circulation during CMV infection. They also show some adaptive features, such as accelerated expansion upon CMV reactivation (29, 30). The main γδ T-cell subset in peripheral blood expresses T-cell receptors encoded by the Vδ2 and Vδ9 gene segments and is referred to as Vδ2^posγδ^ T-cells. γδ T cells that express Vδ1, Vδ3, or Vδ5, but not Vδ2, TCR are collectively designated as Vδ2^negγδ^ T-cells. The latter reside mainly in intestinal and skin epithelia, spleen, and liver (30). Interestingly, a strong and durable expansion of circulating Vδ2^negγδ^ T cells occurs upon CMV reactivation after allo-HSCT (31, 32). This phenomenon is unique for CMV infection and does not occur after infections with other viruses after HSCT (31). Involvement of CMV-induced Vδ2^negγδ^ T cells in protection from leukemic relapse has been suggested by studies showing that expansion of circulating Vδ2^negγδ^ T cells after HSCT is associated with improved leukemia-free survival (33) and that CMV-reactive Vδ2^neg^γδ T cells isolated from CMV-infected HSCT recipients cross-react to primary AML cells (32).

Vδ2^neg^γδ T cells recognize leukemic blasts *via* their TCR, while CD8αα probably serves as a co-receptor in antigen recognition (29). They probably recognize novel antigens in similar manner as Vδ2^posγδ^ T-cells. Quite different from αβ-TCR, Vδ2^posγδ^ TCR recognizes conformational changes in proteins in an antibody-like way (34–36). In addition, natural cytotoxicity receptors, especially NKp30, may play a role in tumor cell recognition by Vδ2^neg^γδ T cells (37).

## Association between CMV Infection and Graft Acceptance in SOT

In experimental animal SOT models, both acute CMV infection and CMV reactivation have been shown to prevent or disrupt graft acceptance, which is thought to result from cross-reactivity of virus-specific T cells to allo-antigens (38). In addition, in the clinical setting, CMV infection after SOT is generally associated with an increased risk of acute and chronic allograft rejection and like for HSCT, CMV infection is a major cause of morbidity and mortality. These data have been summarized in excellent reviews (39–41). Therefore, sophisticated strategies have been implemented to detect CMV early and treat pre-emptively.

However, a main issue in the interpretation of the observed associations between CMV infection and graft rejection is that rejections often occur before CMV infection, and, therefore, it is difficult to prove cause and effect (39, 42). Two recent studies suggest that the link between CMV infection and acute as well as chronic rejection after kidney transplantation are far less significant than previously thought (42, 43). A CMV infection significantly impacts the immune system leaving a clear fingerprint of memory inflation in the T-cell compartment, resembling features of immune aging or senescence (44, 45). This memory inflation is accelerated during CMV reactivation under immunosuppressive medication after SOT (46). Nevertheless, CMV-specific T cells that cross-react to donor cells are only transiently present in the circulation of CMV-infected kidney transplant recipients, and their presence is not associated with inferior graft function (47). Several recent studies even suggest that under certain conditions there may even be an opposite association between CMV infection and graft rejection. First, in elderly kidney transplant recipients, CMV seropositivity was associated with CD4^+^ T-cell immune senescence and freedom of acute rejection (48). Second, donor-specific T-cell hypo-responsiveness, i.e., reduced frequencies of donor-specific, but not of third party-specific T-cells, and reduced immunological graft damage were observed in patients with strong CMV-specific T-cell responses after kidney and heart transplantation (49, 50). Third, increased numbers of terminally differentiated CD8^+^ T-cells, as well as CD4^+^ T cells lacking CD28, both T-cell subsets associated with CMV latency, in the circulation prior to kidney transplantation have been associated with a lower risk for acute rejection (51, 52). Finally, primary CMV infection following LTx is associated with accumulation of terminally differentiated CD8^+^ T cells in the circulation as well as with donor-specific CD8^+^ T-cell hypo-responsiveness and a reduced incidence of acute rejection episodes late after transplantation (53).

As compared with other solid organ grafts, liver grafts display unique immunological features, and LTx is the only setting in which a significant proportion of patients can eventually discontinue immunosuppressive medication without undergoing rejection, a phenomenon known as spontaneous operational tolerance (54). Recent prospective immunosuppression withdrawal studies have shown that operational tolerance can be achieved in about 40% of stable adult LTx patients and 60% of stable pediatric LTX patients (55–57). Interestingly, studies from two different centers found expanded numbers of peripheral Vδ1^+γδ^ T cells and an increased peripheral Vδ1/Vδ2 γδ T-cell ratio in tolerant compared to non-tolerant LTx patients (58–60). In addition, tolerant pediatric LTx recipients exhibit an increased intra-graft Vδ1/Vδ2 ratio (61). A high peripheral Vδ1/Vδ2 γδ T-cell ratio has even successfully been used as a biomarker to select liver transplant (LT) patients for immunosuppression withdrawal (56). Since, durable expansion of circulating Vδ1^+γδ^ T cells and an increased peripheral Vδ1/Vδ2 ratio after CMV infection has been observed in all types of SOT, including LTx (53, 62–65), these observations suggest an association between CMV infection and tolerance after LTx. Such relationship is further supported by a recent study which showed that primary CMV infection after LTx is associated with both expansion of circulating Vδ1^+γδ^ T cells and donor-specific CD8^+^ T cell hypo-responsiveness (53). CMV-responsive Vδ2^−^ γδ T cells have been implicated in antibody mediated rejection after kidney transplant recipients (66), suggesting a different role for these cells after liver transplantation, which may be related to the lower impact of antibodies in liver graft rejection compared to kidney graft rejection.

Increased numbers of circulating terminally differentiated CD8^+^ T cells expressing co-inhibitory receptors is another feature shared by CMV infection (53, 67) and operational tolerance (56) after LTx, again suggesting a possible association. Finally, comparison of gene expression patterns in circulating leukocytes between tolerant and non-tolerant LTx recipients revealed over-expression of NK-cell-related genes in tolerant patients. Interestingly, KLRC4, one of genes encoding NKG2C, which is induced on circulating NK cells by CMV infection and reactivation both after HSCT (20–22, 27) and SOT, including LTx (68–70), was found to be over-expressed in tolerant LTx patients in two different cohorts (59, 71).

Thus, although a direct association between CMV infection and graft acceptance after LTx has not been demonstrated, the presence of sustained immunological imprints of CMV infection in operationally tolerant, but not in non-tolerant, LT patients is strongly suggestive for such association.

## Possible Mechanisms of Graft Acceptance in SOT after CMV Infection

How CMV restrains alloreactivity after LTx remains elusive, and whether CMV-induced peripheral immune cell signatures play a causative role in promoting LT tolerance is as yet unknown. Although Vδ1^+γδ^ T cells under certain conditions may have immune-regulatory properties (72, 73), it is as yet unknown whether they can contribute to liver graft acceptance. However, the massive peripheral expansion of terminally differentiated CD8^+^ T cells expressing co-inhibitory receptors upon CMV infection after LTx may contribute to reduced T-cell alloreactivity to organ grafts, since these cells show impaired functional responses to allo-antigens (53, 67) (Figure 1). In addition, as terminally differentiated CD8^+^ T cells poorly infiltrate in organ grafts, they might not contribute to graft rejection even when functionally competent to respond to allo-antigens (53, 74).

Cytomegalovirus infection of the liver graft may also promote resistance of liver grafts to allogeneic attack by triggering type I interferon production (75) and recruitment of T-helper 1 cells that produce IFN-γ (76), which is strikingly absolutely needed for LT tolerance in animal models (77). IFN-γ produced by graft-infiltrating T cells critically contributes to immunological tolerance of liver grafts in experimental animals by induction of intra-graft PD-L1 expression that leads to T-cell apoptosis (78). Indeed, interaction of PD-L1 expressed in the liver graft with the co-inhibitory receptor PD-1 on graft-infiltrating T cells also counter-regulates rejection activity against liver grafts in humans (79) (Figure 1). Although this mechanism of CMV-driven graft anti-host resistance is speculative, a role for intra-graft interferon signaling, in the development of operational tolerance after LTX has been suggested by an immunosuppression withdrawal trial in HCV-infected LT patients in which operationally tolerant patients were observed to overexpress interferon-stimulated genes as well as PD-L1 and PD-1 in their liver graft (56). Such so-called “adaptive immune resistance” mechanisms, in which expression of immunosuppressive molecules, such as PD-L1, is induced in response to IFN type I and/or IFN-γ produced by infiltrating immune cells, are also utilized by tumors to escape immune attack (80). In addition, recent data show that IFN-type I signaling during chronic viral infections may promote CD8^+^ T cell exhaustion and impair memory T-cell responses against unrelated antigens (81, 82), connecting IFN-signaling to terminal differentiation and exhaustion of CD8^+^ T cells.

Finally, one or more of the well-established immune evasion strategies of CMV to establish latency may be involved, such as production of viral IL-10, which may exert systemic immunosuppressive effects (83).

## Concluding Remarks

Cytomegalovirus infection, although generally disadvantageous in immunosuppressed subjects, paradoxically has beneficial effects in HSCT and probably also in LTx recipients. More research is definitely needed to substantiate and better understand these enigmatic observations. Whereas, the anti-leukemic effect of CMV infection after HSCT in AML patients is firmly established, prospective studies are required to investigate whether there is a direct association between CMV infection after LTx and development of allogeneic liver graft acceptance. Such studies should be accompanied by extensive peripheral and intra-graft immune profiling to begin to understand the putative immunological mechanisms linking CMV infection with tolerance after LTX. Similarly, studies aiming to further unravel the immunological mechanisms linking CMV infection and prevention of AML recurrence after HSCT are required. Once those benefits and the underlying mechanisms have been firmly established, development of therapeutic approaches to mimic the beneficial effects of CMV infection after HSCT for AML and after LTx becomes an interesting, although challenging aim. Development of a CMV vaccine that induces similar immunological imprints as CMV infections might be a way to achieve this goal.

## Author Contributions

NL, LW, JKu, and JKw all participated in writing the manuscript. NL and JKw co-edited the final version of the manuscript. All authors have read and approved the final manuscript.

## Conflict of Interest Statement

NL, LW, and JKw have nothing to disclose. JKu reports grants from Novartis and Miltenyi Biotech. He is scientific co-founder and CSO of gadeta (www.gadeta.nl) and inventor on multiple patents on γδ TCR receptors and isolation strategies for engineered immune cells.

## Abbreviations

CMV, cytomegalovirus; HSCT, hematopoietic stem cell transplantation; AML, acute myeloid leukemia; SOT, solid organ transplantation; LT patient, liver transplant patient; LTx, liver transplantation; ALL, acute lymphoblastic leukemia (ALL); CML, chronic myeloid leukemia; MDS, myelo-dysplastic syndrome (MDS); a/cGVHD, acute/chronic graft versus host disease; KIR, killer cell immunoglobulin-like receptors.
